# Spontaneous capsular rupture and dislocation of the lens nucleus into the anterior chamber in Morgagnian cataract: A case report

**DOI:** 10.1097/MD.0000000000049228

**Published:** 2026-06-12

**Authors:** Yoshitaka Orio, Yoshinari Saima, Yugo Hiranuma, Hidenobu Asami, Yoshiaki Tanaka, Akihiro Kakehashi, Toshikatsu Kaburaki, Suguru Nakagawa

**Affiliations:** a Department of Ophthalmology, Saitama Medical Center, Jichi Medical University, Saitama City, Saitama, Japan.

**Keywords:** extracapsular cataract extraction, hypermature cataract, lens nucleus dislocation, Morgagnian cataract, spontaneous capsular rupture

## Abstract

**Rationale:**

Morgagnian cataracts represent a hypermature stage of cataract formation characterized by cortical liquefaction and a sunken nucleus. Spontaneous rupture of the lens capsule is extremely rare but can result in dislocation of the lens nucleus into the anterior chamber.

**Patient concerns:**

A 75-year-old woman presented with mild ocular pain and decreased vision in her left eye for 1 week. She had undergone pars plana vitrectomy for retinal detachment approximately 35 years earlier.

**Diagnoses:**

Slit-lamp biomicroscopy revealed a dense brown lens nucleus dislocated into the anterior chamber with evidence of spontaneous capsular rupture. B-scan ultrasonography showed no retinal detachment.

**Interventions:**

Lens nucleus extraction combined with pars plana vitrectomy was performed without intraocular lens implantation. The lens nucleus, located in the anterior vitreous cavity at the beginning of surgery, was elevated onto the iris surface using a light guide and a Sinskey hook and subsequently removed through a superior corneoscleral incision.

**Outcomes:**

Postoperatively, the eye remained quiet with stable intraocular pressure, although visual recovery was limited (0.04) because of preexisting chorioretinal atrophy.

**Lessons:**

This case highlights a rare occurrence of spontaneous capsular rupture and dislocation of the lens nucleus in a Morgagnian cataract following vitrectomy. Long-term zonular and capsular weakening after vitrectomy may predispose to delayed rupture. Early cataract surgery and regular follow-up are essential to prevent spontaneous rupture and subsequent lens-induced inflammation and glaucoma in hypermature cataracts.

## 1. Introduction

Morgagnian cataract is a type of hypermature cataract in which cortical fibers become liquefied, causing the dense brown nucleus to sink inferiorly within the capsular bag.^[1]^ In rare instances, spontaneous rupture of the lens capsule may occur, leading to the dislocation of the lens nucleus into the anterior chamber or vitreous cavity.^[2–10]^ We report a rare case of Morgagnian cataract with spontaneous capsular rupture and anterior dislocation of the lens nucleus into the anterior chamber in a patient with a history of vitrectomy.

## 2. Case report

The requirement for approval of this case report was waived by the ethics committee at Jichi Medical University owing to the retrospective nature of the study. This case report adheres to the Declaration of Helsinki. Written informed consent for publication was obtained.

The patient was a 75-year-old woman who experienced mild pain in the left eye for 1 week in late August of the index year. She had a history of hypertension and retinal detachment in the left eye (pars plana vitrectomy approximately 35 years earlier). She was diagnosed with anterior dislocation of the lens by a local ophthalmologist and was referred to our hospital for further evaluation.

### 2.1. Initial examination

**Figure 1.**
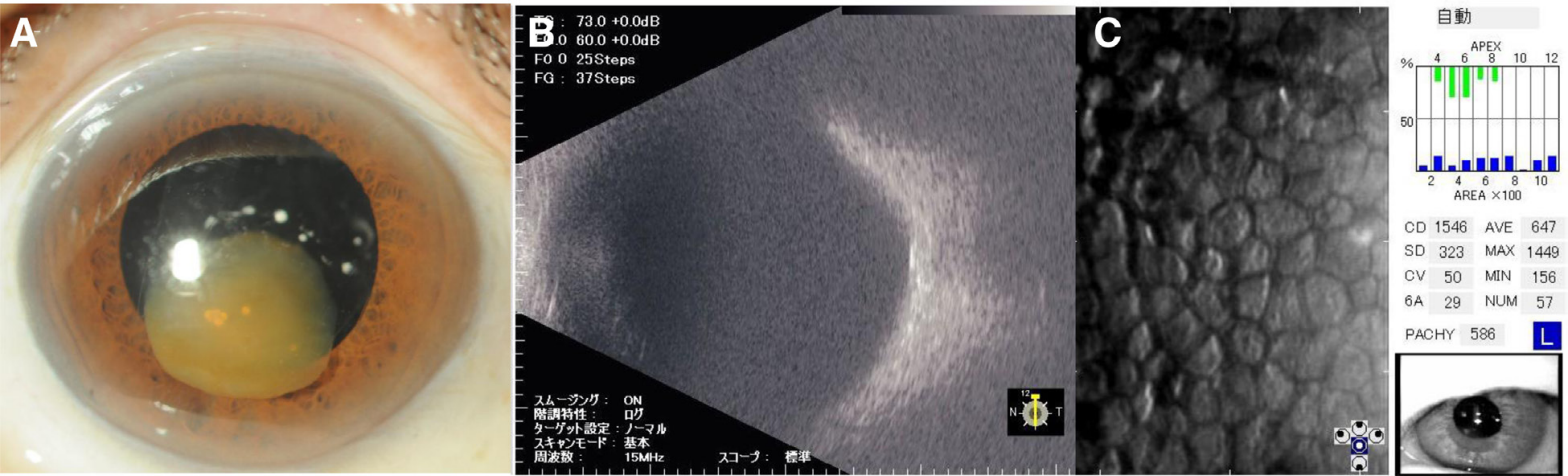
Findings at the initial examination in our department. (A) Dislocated lens nucleus in the anterior chamber. (B) B-scan ultrasonography showing no retinal detachment. (C) Specular microscopy at the initial visit showing mildly reduced corneal endothelial cell density (1546 cells/mm^2^ in the left eye).

Best-corrected visual acuity was 0.8 in the right eye and limited to hand motion in the left eye. The intraocular pressure (IOP) was 11 mm Hg (right) and 16 mm Hg (left). Endothelial cell density was 2004 and 1546 cells/mm^2^ in the right and left eyes, respectively, indicating mild endothelial loss. Gonioscopy revealed open angles (Shaffer grade 4) in both eyes, without peripheral anterior synechiae. Slit-lamp biomicroscopy revealed a moderately advanced cataract in the right eye and a brown dense lens nucleus dislocated into the anterior chamber of the left eye, suggesting spontaneous capsular rupture (Fig. 1). Although the fundus view was poor, B-scan ultrasonography revealed no retinal detachment. Based on these findings, spontaneous rupture of the lens capsule with dislocation of the nucleus into the anterior chamber was diagnosed.

### 2.2. Treatment and clinical course

**Figure 2.**
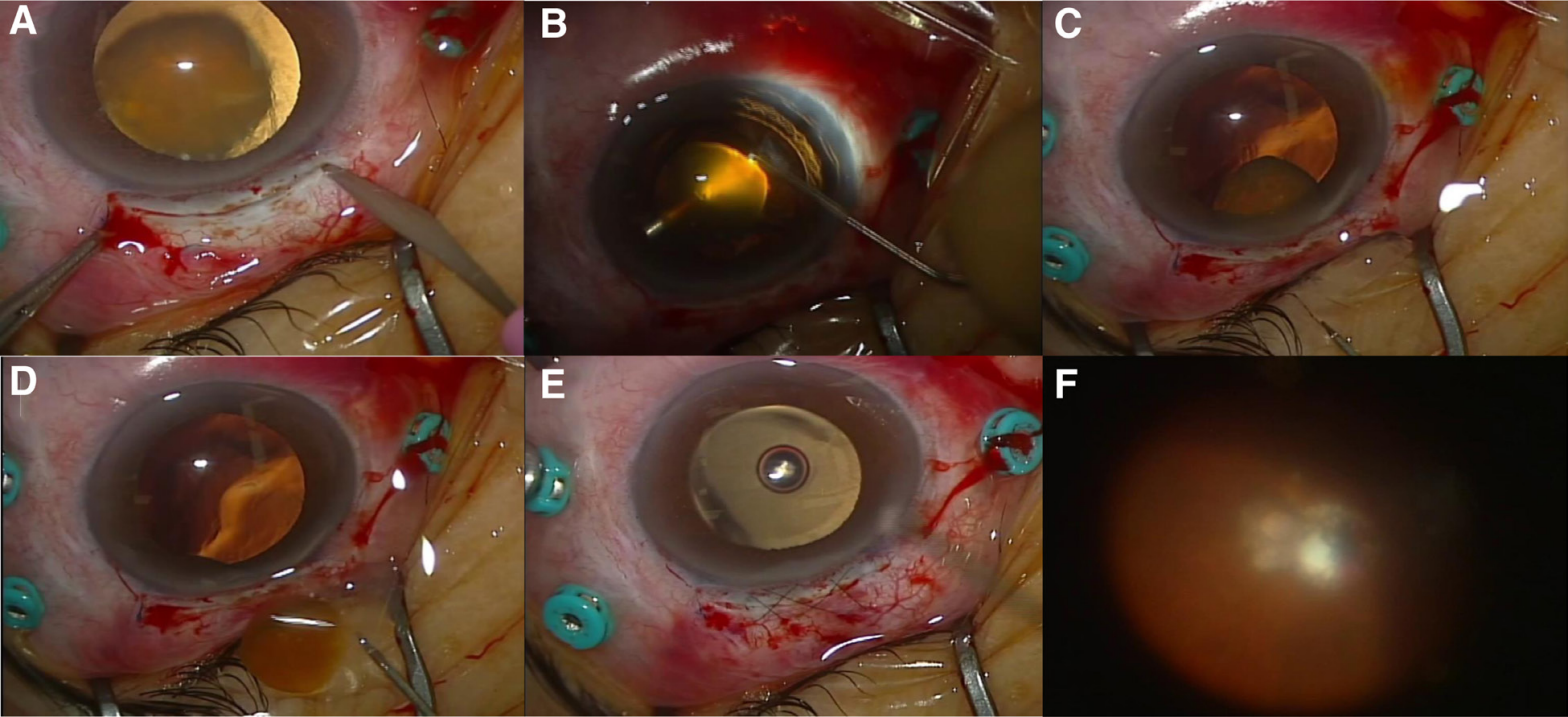
Intraoperative findings. (A) Tri-port 25G vitrectomy setup with a 7-mm corneoscleral incision. Three 25G trocars are inserted 3.5 mm posterior to the limbus, and a 7-mm superior corneoscleral incision is created. (B) Elevation of the lens nucleus using a 25G light guide and Sinskey hook. (C and D) Extraction of the capsule and lens nucleus. The lens nucleus was removed together with the capsule using a viscoextraction technique. When the residual capsule was gently grasped and withdrawn with forceps, the nucleus was subsequently extracted through the incision, following the capsule. The viscoelastic material facilitated this viscoextraction process. (E) Closure of the main incision. (F) Confirmation of extensive chorioretinal atrophy.

In September of the same year, the patient underwent lens nucleus extraction combined with a pars plana vitrectomy of the left eye using a 25-gauge Constellation® Vision System (Alcon Laboratories; Fig. 2 and Video S1, Supplemental Digital Content). Although the lens nucleus had been located in the anterior chamber on initial examination, it was found in the anterior vitreous cavity at the beginning of surgery. We speculate that preoperative pupillary dilation and the positional change from sitting to supine allowed the highly mobile nucleus to migrate posteriorly into the anterior vitreous cavity. After administering sub-Tenon’s anesthesia with 2% lidocaine, three 25-gauge trocars were inserted 3.5 mm posterior to the limbus, and a 7-mm superior corneoscleral incision was made. After injecting viscoelastic material into the anterior chamber, the dislocated lens nucleus was elevated onto the iris surface using a light guide and a Sinskey hook. Additional viscoelastic material was then injected posterior and inferior to the lens nucleus to advance it toward the incision by the viscoextraction technique, although complete extraction through the wound was not achieved at this stage.

Subsequently, the residual capsule was gently grasped with forceps and withdrawn, during which the lens nucleus was extracted through the incision following the capsule. The viscoelastic material facilitated this extraction process.

Triamcinolone acetonide was injected to visualize the residual vitreous, which was found to be minimal. The prior vitrectomy performed 35 years earlier had likely removed most of the vitreous cortex. Extensive chorioretinal atrophy, which had been obscured preoperatively by the dense dislocated lens nucleus, was revealed during surgery. After confirming the absence of retinal tears or holes, the surgery was completed without intraocular lens (IOL) implantation.

**Figure 3.**
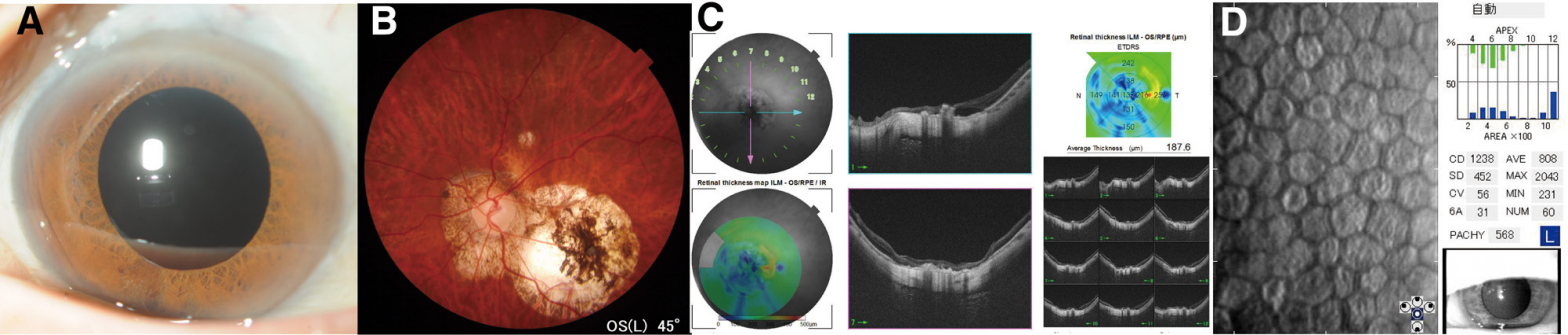
Findings at the final follow-up (6 months postoperatively). (A) Anterior segment at the final visit. (B) Fundus photograph showing marked chorioretinal atrophy. (C) OCT image showing macular chorioretinal atrophy. (D) Specular microscopy at the final visit showing a corneal endothelial cell density of 1238 cells/mm^2^, representing approximately a 20% decrease compared with the preoperative value. OCT = optical coherence tomography.

Six months postoperatively (February of the following year), visual acuity in the left eye remained poor (0.04) due to extensive chorioretinal atrophy that had been obscured preoperatively by the dense dislocated lens nucleus (Fig. 3). However, the IOP remained stable at 13 mm Hg without any signs of inflammation, and the overall postoperative condition of the eye was stable (Fig. 3). Follow-up at our institution was completed at 6 months, after which the patient was referred back to the local ophthalmologist for continued management.

## 3. Discussion

We encountered a rare case of spontaneous rupture of the anterior lens capsule in a patient with Morgagnian cataract that developed after vitrectomy for retinal detachment, leading to liquefaction of the cortical material and anterior dislocation of the dense nucleus.

In 1769, the anatomist Giovanni Battista Morgagni described hypermature cataracts in his work, *The Seats and Causes of Diseases Investigated by Anatomy*, and the condition was later named after him.^[11]^ In Morgagnian cataracts, the liquefaction of the cortex causes the nucleus to sink inferiorly, resulting in a characteristic appearance.^[1]^ Although spontaneous rupture of the anterior or posterior capsule with nuclear dislocation has been described, such cases are extremely rare (Table 1).

**Table 1:** Reported cases of spontaneous capsular rupture and lens nucleus dislocation in hypermature (Morgagnian) cataract.

Author (yr)	Age (yr)	Sex	Findings	Medical history	IOP (pre/post, mm Hg)	BCVA (pre/post, decimal)	Fellow-eye BCVA
Ballen (1955)	70	M	Anterior rupture, anterior dislocation	None	**60**/20	LP/0.6	0.8
Ming (1963)	50	F	Anterior rupture, anterior dislocation	None	Normal/NA	LP/CF	HM (dense mature cataract)
Hemalatha (2012)	52	F	Anterior rupture, anterior dislocation	None	12/NA	0.1/no surgery	0.1
Nishino (2013)	65	F	Anterior rupture, anterior dislocation	Glaucoma	**76**/normal	HM/HM	0.2
Malik (2014)	50	F	Posterior rupture, posterior dislocation	None	12/NA	0.5/0.3	NA
Petrovic Pajic (2020)	79	F	Posterior rupture, posterior dislocation	Macular hole	High/normal	0.1/0.7	NA
Bievel-Rădulescu (2021)	83	M	Anterior rupture, anterior dislocation, hypopyon	None	**50**/17	LP/0.5	1.0
Guan (2022)	50	M	Anterior rupture, anterior dislocation	None	8/13	CF/0.05	1.0
Guan (2022)	76	M	Anterior rupture, anterior dislocation	Glaucoma	**32**/17	CF/0.05	0.4
Yu (2024)	74	F	Anterior rupture, anterior dislocation	None	14/13	LP/LP	0.6
Orio (2025)	75	F	Anterior rupture, anterior dislocation	After vitrectomy for RD; chorioretinal atrophy	16/13	HM/0.04	0.8

Values in bold indicate elevated IOP. “Pre” and “post” denote preoperative and postoperative measurements, respectively.

BCVA = best-corrected visual acuity (decimal), CF = counting fingers, HM = hand motion, IOP = intraocular pressure, LP = light perception, NA = not available, RD = retinal detachment.

A PubMed search using the terms “hypermature cataract” and “spontaneous rupture” (August 27, 2025) yielded 13 results. The major published cases after excluding nonhuman and image-only reports are summarized in Table 1.

In the present case, the dislocated nucleus was removed through a 7-mm superior corneoscleral incision. The nucleus of a Morgagnian cataract is extremely hard and cannot be adequately fragmented with a vitreous cutter; therefore, either phacoemulsification using an ultrasonic tip or extracapsular extraction through a corneoscleral incision is required. Because the lens capsule provided no support, phacoemulsification carried a high risk of vitreoretinal traction and re-drop of the nucleus into the posterior cavity. Therefore, extracapsular extraction via a corneoscleral incision was chosen.

The size of the nucleus allowed smooth removal through the 7-mm incision without intraoperative complications. Postoperatively, mild corneal endothelial cell loss was observed. Considering that the preoperative endothelial cell density was already reduced to approximately 1546 cells/mm^2^ and that the extracted nucleus was extremely hard due to an advanced Morgagnian cataract, the postoperative reduction to approximately 1238 cells/mm^2^ (≈20%) was considered acceptable and within the clinically tolerable range.

In this case, IOL implantation was not performed because of high myopia and extensive chorioretinal atrophy. If IOL implantation had been considered, scleral fixation^[12–17]^ would have been required, as capsular support could not be expected. Sutureless intrascleral fixation^[12–14,16]^ is particularly advantageous for eyes lacking capsular support; it prevents suture-related complications and offers a useful option for cases of spontaneous capsular rupture such as this one.

## 4. Possible mechanisms

Several factors may contribute to spontaneous rupture. Age-related degeneration increases intralenticular pressure as the cataract progresses, while zonular fibers degenerate, and the capsule becomes calcified. Increased capsular permeability may allow the liquefied cortex to leak into the anterior chamber or vitreous cavity, gradually weakening the capsule until rupture and nuclear dislocation occur.^[1]^

In this case, a previous vitrectomy likely played an additional role. Long-term mechanical or gas-tamponade–related stress after vitrectomy can cause posterior capsular thinning or instability, possibly leading to delayed rupture.^[18]^ Instrumental contact or traction of the zonular fibers during anterior vitrectomy may also weaken the lens capsule.

Among previous reports of spontaneous rupture in hypermature cataracts (Table 1), some were complicated by intraocular inflammation or secondary glaucoma, but none involved patients who underwent prior vitrectomy. In a series by Goel et al involving 10 eyes with spontaneous capsular rupture, none of the patients had a history of vitrectomy. In that series, 3 eyes exhibited anterior and 2 posterior dislocations of the nucleus, all with an absorbed cortex, similar to our case. Only 1 eye had intraocular inflammation and elevated IOP. An IOL was successfully implanted in 7 of the 10 eyes, and the postoperative visual acuity ranged from 0.15 to 0.3.^[19]^ Notably, all fellow eyes had previously undergone successful cataract surgery and had good vision. The authors suggested that the delayed treatment of the affected eye was related to economic constraints and adequate vision in the fellow eye. Similarly, our patient had good vision in the right eye and had “given up” on her left eye due to prior retinal detachment, resulting in decades without ophthalmic follow-up.

Spontaneous rupture of the capsule can lead to severe complications such as phacolytic glaucoma and lens-induced uveitis caused by the leakage of lens proteins and macrophage accumulation in the trabecular meshwork.^[20]^ Therefore, regular ophthalmologic follow-up and timely cataract surgery are crucial for preventing secondary complications.

## Acknowledgments

We thank Editage (www.editage.com) for the English language editing.

## Author contributions

**Conceptualization:** Suguru Nakagawa.

**Writing – original draft:** Yoshitaka Orio, Suguru Nakagawa.

**Data curation:** Yoshitaka Orio, Suguru Nakagawa.

**Writing – review & editing:** Yoshinari Saima, Yugo Hiranuma, Hidenobu Asami, Yoshiaki Tanaka, Akihiro Kakehashi, Toshikatsu Kaburaki, Suguru Nakagawa.

**Supervision:** Akihiro Kakehashi, Toshikatsu Kaburaki, Suguru Nakagawa.

**Formal analysis:** Suguru Nakagawa.

**Funding acquisition:** Toshikatsu Kaburaki, Suguru Nakagawa.

**Investigation:** Suguru Nakagawa.

**Methodology:** Suguru Nakagawa.

**Project administration:** Suguru Nakagawa.



## References

[1] Bron AJ Habgood JO. Morgagnian cataract. Trans Ophthalmol Soc U K (1962). 1976;96:265–77. 1070881

[2] Bievel-Rădulescu R Tăbăcaru B Stanca HT. Lens-induced uveitis in a patient with hypermature cataract. Rom J Ophthalmol. 2021;65:300–6. 35036658 10.22336/rjo.2021.62 PMC8697789

[3] Guan JY Ma YC Zhu YT. Lens nucleus dislocation in hypermature cataract: case report and literature review. Medicine (Baltimore). 2022;101:e30428. 36107580 10.1097/MD.0000000000030428 PMC9439833

[4] Hemalatha C Norhafizah H Shatriah I. Bilateral spontaneous rupture of anterior lens capsules in a middle-aged woman. Clin Ophthalmol. 2012;6:1955–7. 23225999 10.2147/OPTH.S37276 PMC3514058

[5] Malik VK Jhalani R Malik KP Gupta A. Spontaneous rupture of lens capsule with dislocation of nucleus in hypermature cataract. Nepal J Ophthalmol. 2014;6:95–7. 25341832 10.3126/nepjoph.v6i1.10778

[6] Yu X Chen X-H Dai Y. Spontaneous dislocation of the lens nucleus into the anterior chamber observed in a patient with overmature senile cataract. J Craniofac Surg. 2024;35:e463–6. 38781430 10.1097/SCS.0000000000010293

[7] Nishino K Yoshida T Nitta A Saito S Saito H. A case of Morgagnian cataract complicated by phacolytic glaucoma. Rinsho Ganka (Jpn J Clin Ophthalmol). 2013;67:1078–81.

[8] Ballen PH Hughes WL. Spontaneous rupture of lens capsule in hypermature (Morgagnian type) cataract. Am J Ophthalmol. 1955;39:403–5. 14350057 10.1016/0002-9394(55)91288-5

[9] Ming AL. Spontaneous rupture of the anterior capsule of a hypermature lens. Singapore Med J. 1963;3:127–30. 14075737

[10] Petrovic Pajic S Lumi X Schollmayer P Hawlina M. Spontaneous full thickness macular hole development and closure in a patient with nucleus dislocation due to hypermature cataract: a case report. Croat Med J. 2020;61:366–70. 32881435 10.3325/cmj.2020.61.366 PMC7480756

[11] Knapp A. Observations on glaucoma in Morgagnian cataract. Trans Am Ophthalmol Soc. 1926;24:84–92. 16692750 PMC1316563

[12] Nakagawa S Kanda S Ishii K. Secondary intrascleral intraocular lens fixation with lens capsule preservation for aphakic eyes in patients with pseudoexfoliation syndrome: a case series. Cureus. 2024;16:e70688. 39372382 10.7759/cureus.70688 PMC11452084

[13] Nakagawa S Totsuka K Okinaga K Takamoto M Ishii K. Background factors determining the time to intraocular lens dislocation. Int Ophthalmol. 2024;44:240. 38904711 10.1007/s10792-024-03166-x

[14] Nakagawa S Ishii K. Secondary intrascleral intraocular lens (IOL) fixation with capsule preservation for IOL dislocation following mature cataract surgery with incomplete capsulorhexis: a case report. Medicine (Baltimore). 2025;104:e43030. 40550021 10.1097/MD.0000000000043030 PMC12187320

[15] Nakagawa S Okubo A Ishii K. Resuturing a dislocated scleral-fixated intraocular lens in brown-McLean syndrome. J Clin Med. 2025;14:5769. 40869595 10.3390/jcm14165769 PMC12387824

[16] Yamane S Sato S Maruyama-Inoue M Kadonosono K. Flanged intrascleral intraocular lens fixation with double-needle technique. Ophthalmology. 2017;124:1136–42. 28457613 10.1016/j.ophtha.2017.03.036

[17] Chan CC Crandall AS Ahmed IIK. Ab externo scleral suture loop fixation for posterior chamber intraocular lens decentration: clinical results. J Cataract Refract Surg. 2006;32:121–8. 16516790 10.1016/j.jcrs.2005.06.050

[18] Imen K Meriam BHT Ilhem S Sonia A Nesrine A Moncef K. Lens-induced hypopyon uveitis as the presenting manifestation of posterior lens nucleus dislocation following pars-plana vitrectomy: case report. J Ophthalmic Inflamm Infect. 2021;11:42. 34783918 10.1186/s12348-021-00273-z PMC8595433

[19] Goel N Nagar M. Spontaneous rupture of the lens capsule in hypermature cataract: presentations and outcomes. Br J Ophthalmol. 2016;100:1081–6. 26567023 10.1136/bjophthalmol-2015-307184

[20] Irvine SR Irvine AR Jr. Lens-induced uveitis and glaucoma. III. “Phacogenetic glaucoma”: lens-induced glaucoma; mature or hypermature cataract; open iridocorneal angle. Am J Ophthalmol. 1952;35:489–99. 14914837

